# Addressing limited weight resolution in a fully optical neuromorphic reservoir computing readout

**DOI:** 10.1038/s41598-021-82720-4

**Published:** 2021-02-04

**Authors:** Chonghuai Ma, Floris Laporte, Joni Dambre, Peter Bienstman

**Affiliations:** 1 Photonics Research Group, UGent - imec, Technologiepark-Zwijnaarde 126, 9052 Ghent, Belgium; 2 IDLab, UGent - imec, Technologiepark-Zwijnaarde 126, 9052 Ghent, Belgium

**Keywords:** Optics and photonics, Electrical and electronic engineering, Computer science

## Abstract

Using optical hardware for neuromorphic computing has become more and more popular recently, due to its efficient high-speed data processing capabilities and low power consumption. However, there are still some remaining obstacles to realizing the vision of a completely optical neuromorphic computer. One of them is that, depending on the technology used, optical weighting elements may not share the same resolution as in the electrical domain. Moreover, noise of the weighting elements are important considerations as well. In this article, we investigate a new method for improving the performance of optical weighting components, even in the presence of noise and in the case of very low resolution. Our method utilizes an iterative training procedure and is able to select weight connections that are more robust to quantization and noise. As a result, even with only 8 to 32 levels of resolution, in noisy weighting environments, the method can outperform both nearest rounding low-resolution weighting and random rounding weighting by up to several orders of magnitude in terms of bit error rate and can deliver performance very close to full-resolution weighting elements.

## Introduction

Machine learning is becoming ubiquitous in people’s daily lives. It can achieve outstanding performance on a variety of tasks^1–3^. However, the surge of the vast volumes of generated data is approaching the limits of the conventional Von Neumann architectures in electrical hardware, as Moore’s law appears to be coming to an end. Optical neuromorphic computing^4–8^, with its high-efficiency high-speed data processing capabilities, is becoming more and more a viable choice as a hardware implementation for neural networks, especially for problems with high data volumes where the input is already in the optical domain. Integrated silicon photonics neuromorphic systems have proven themselves as potential options, especially since they can exploit CMOS fabrication technology and are therefore suited for mass production^9^.

### Photonic reservoir computing

**Figure 1:**
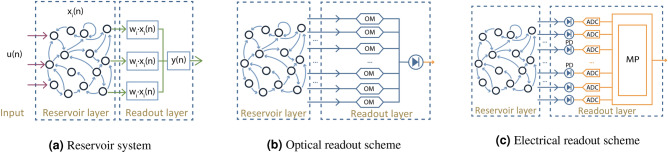
(**a**) Reservoir computing consists of a reservoir layer, which is inherently an untrained RNN, and a readout layer that weights and linearly combines of output channels of the reservoir. (**b**) An integrated optical readout takes advantage of standard optical modulators (OM) to weigh the optical signals from the reservoir in both amplitude and phase with no latency and very low energy cost. The linear combination of the optical signals can be achieved using optical combiners. (**c**) A Conventional integrated electrical readout requires an individual photodetector (PD) and Analog-To-Digital converter (ADC) for each output channel, as well as a microprocessor (MP) to perform the linear combination of the signals, which introduces unwanted latency and power consumption.

Reservoir computing (RC) is one of the machine learning paradigms^10,11^ whose relaxed requirements make it well suited for a hardware implementation. A reservoir is inherently a randomly initialized, untrained recurrent neural network (RNN), which acts as a temporal prefilter to transform a time dependent input into a higher-dimensional space, where it can be more easily classified by a linear classifier. Therefore, unlike RNNs, reservoir computing does not rely on optimizing the internal interconnection parameters of the network. Instead, it only optimizes the weights of the linear combination in the readout layer as shown in Fig. 1a. Because the performance of the reservoir is, within certain bounds, relatively insensitive to the exact internal weights of the reservoir, this technology is very interesting for a (photonic) hardware implementation, where fabrication tolerances are inevitable. Specific to photonic implementations are the so-called passive reservoirs, where the reservoir itself does not contain any nonlinearity, but where the photodetector in the readout (which converts a complex-valued light amplitude to a real-valued power intensity) provides the required non-linearity. Doing away with the need for internal nonlinearities reduces the power consumption. Photonic integrated circuits like these have been reported in^4,12–14^, solving parity bit tasks, header recognition and telecom signal regeneration tasks at data rates around 10 Gb/s. Laporte et al.^5,15^ use photonic crystal mixing cavities as the reservoir and also could solve the XOR task on neighboring bits, with the potential of achieving much higher speeds, theoretically up to several hundreds of Gb/s.

In the meantime, other neuromorphic computing approaches are gaining prominence. E.g. optical feed-forward neural networks for deep learning are proposed by Shen et al.^7^, which cascade integrated coherent optical matrix multiplication units embedded in programmable nanophotonic processors. The results show its utility for vowel recognition. As another approach, diffractive optical machine learning^8^ is achieved using diffractive elements as interconnection layers, which can solve various computational functions. A multiple-wavelength-based system is presented in^16^.

### Readout system

To fully exploit a coherent optical neuromorphic computing circuit, thus benefiting from its high data rates and low energy consumption, an integrated optical readout to calculate the weighted sum (Fig. 1b) is preferable. The critical aspect for such an optical readout is that the weights are applied in the optical domain, instead of converting optical signals to electrical signals, and then applying the weights on the electrical signals using a microcomputer (referred as electrical readout schemes as shown in Fig. 1c). Apart from speed, latency, and power efficiency advantages, such a fully optical readout system can also perform weighting on phase and amplitude separately, which results in more degrees of freedom, and thus more computation capability.

One issue of an optical readout is that there is no longer an explicit electronic observability for each of the states, since in such systems it is desirable to only have one single photodiode and high-speed analog-to-digital converter after the linear combination. This observability problem was dealt with by Freiberger et al.^17^.

Another problem is that, depending on the technology, the resolution of the optical weights could be much lower compared to applying weights in the electrical domain, which can easily reach 16 bit and more. For a photonic reservoir with optical readout illustrated as Fig. 1b, the situation can be different depending on the weights are physically implemented. One option for the OM (optical modulator) is a heater-based optical interferometer. By driving the heater current to produce temperature changes on an integrated waveguide, the phase and amplitude of the optical signal can be modified. In this case, the resolution of the current source will be the limitation of the weighting resolution and the weights will be quantized by the resolution of the DAC that drives the current source. So, in this case, weight resolution will not be a problem, but this heater-based implementation consumes a lot of power and is volatile.

Another more extreme example is a Barium Titanate (BTO)^18^ weighting element, which has the critical advantage of being non-volatile, consuming nearly zero power while weighing the signal. Unlike the heater-based OM, we control the BTO material properties by programming it with a voltage, instead of changing the temperature. However, due to the stability of the ferroelectric properties of the material, there are only very few levels available for adjustment, therefore causing a very low resolution on the order of 3–5 bit (10–30 levels). Additionally, this is coupled with some inevitable noise due to potential parameter drifting of the optical elements.

In this paper, we will consider the extreme example of a BTO weighting element and explore strategy to solve the low weighting resolution problem in a general way.

### Weight quantization

Network quantization is a important topic for deep learning researchers and developers, since the demand for applying machine learning tasks on low power consumption systems, e.g. mobile devices, has been increasing drastically^19^. However, almost all of the methods and algorithms have a focus on existing electrical hardware, like GPU or CPU. As hardware photonic neuromorphic computing networks have been thriving recently, and since the weighting method is completely different, it needs its own quantization strategy that suits its features and maximizes the performance from optical weighting elements. The differences are reflected in the following aspects.

#### Weighting resolution

First, for deep learning network quantization in the electrical domain, the target resolution is mostly based on the application hardware. Vanhoucke et al.^20^ implemented an 8-bit integer resolution activation instead of 32-bit floating-point to optimize CPU performance. Courbariaux et al.^21^ explored binary weights to accelerate future dedicated low-power electrical deep learning hardware by replacing multiply-accumulate operations by simple accumulations, which is called BinaryConnect method. However, for photonics, getting rid of multiplications is not so relevant, as these can be done in the analog optical domain instantly anyhow. The BinaryConnect method was later extended to a power-of-two setting by the Gudovskiy et al.^22^, but even then, the peculiarities of photonic hardware are such that a new discretisation strategy is required. Indeed, in integrated photonics systems weighting is usually achieved by controlling on-chip heaters or non-volatile materials in an optical interferometer to modulate the amplitude or phase of the coherent optical signal. Depending on the technology, the resolution varies between 3 and 6 bit, but more importantly, it is often impossible to achieve a weight identical to zero. This aspect needs to be accounted for explicitly, and hence a new discretization strategy is required.

#### Number of parameters

Most deep neural networks have a substantial number of trainable parameters; some of them may reach tens of millions, leading to a significant redundancy in deep learning models^23^. Network pruning is often a feasible option for reducing the size of the network in parallel to quantization. However, for optical systems like passive reservoir computing chips, the number of trainable parameters in the readout system is typically in the hundreds or fewer, which leads to a reduced parameter redundancy and therefore a potentially much more severe impact on the performance by the weight quantization. On the other hand, each trainable parameter is corresponding to a real optical channel, which also makes pruning not possible, since it would result in additional optical loss.

#### Quantization details

As mentioned above, weighting in a coherent photonic neuromorphic hardware is different compared to weighting in a CPU or GPU. One of the differences lies in the nature of the quantized weight values. In^21^, the quantized weights can be chosen from 1 or − 1, and for the majority of deep learning networks, there is no restriction on the distribution of the weight values. However, for amplitude weighting in the coherent photonic neuromorphic hardware, it is not possible to achieve a weight value of exactly 0, due to inevitable hardware imperfection. As a example, an optical interferometer has a ’on’ state and an ’off’ state, but because of fabrication imperfections causing loss imbalance, the ratio between the intensity of the ’on’ state and the intensity of the ’off’ states can never reach infinity. This ratio is so-called extinction ratio of highest to lowest weight, which is an important implementation aspect. For phase weighting on the other hand, there is no such restriction, and the values can range from 0 to $2\pi$.

#### Noise

Another difference is that for quantization in a CPU- or GPU-based deep networks, since they are digital systems, there should be negligible noise on the quantization weights. Although some networks apply noise on the weights to improve the robustness of the system^24^, the added noise is often really small compared to the quantization interval. In contrast, in hardware optical weighting elements, noise is inevitable, and can sometimes be significant, which represents additional challenges.

Due to the many intrinsic differences between a photonic weighting system and an electrical weighting mechanism as discussed above, it is very difficult to apply a deep learning quantization method directly to photonic hardware. In such a context, it is of great importance to train a readout system for coherent optical neuromorphic computing systems, so that the performance is less sensitive to the quantization and the noise of the weights. This is the exact problem we want to tackle in this paper.

The rest of this paper is structured as follows. In “Methods” section, we introduce our modeling method of the optical weighting hardware, in amplitude and phase, by taking into consideration the number of quantization levels, the extinction ratio of the elements, and the noise after quantization. The explorative quantization retraining technique used in this work will also be highlighted. In “Results” section, we show our simulation results based on our quantization method with respect to the different hardware parameters mentioned in “Methods” section. We also discuss the quantization result for various tasks.

## Methods

In this part, we introduce the concept of our quantization method and present the implementation details like the discretization model, the type of regularization and the training procedure.

### Explorative quantization weight selection

The explorative quantization weight selection in this work is inspired by methods that have been used in deep learning quantization. Typically, after a full precision model has been trained, a subset of weights is identified to be either pruned^25^ or kept fixed^26^. The other weights are then retrained in full precision and requantized. If necessary, this step can be repeated in an iterative fashion, retraining progressively smaller subsets of the weights in order to find the most optimal and stable solution.

A crucial part of these methods is selecting a subset of weights to be left fixed or to be pruned. Random selection of weights is not a good idea, because there is a high probability of eliminating ‘good’ weights that convey important information. Han et al.^25^ and Zhou et al.^26^ tackle this problem by choosing the weights with the smallest absolute value.

This is reasonable in deep learning models, since the millions of weights can provide enough tolerance when it comes to accidentally selecting the ‘wrong’ weights. However, in the readout systems we are investigating here, we have much fewer weights and a much more limited resolution with severe noise. In this case, the absolute value will not provide enough information, as a combination of many small weights could be important in fine-tuning the performance of the network. This will lead to a risk of a huge accuracy loss when specific ‘wrong’ connections (that are more sensitive to perturbations) are chosen to be retrained.

Instead, we adopt a different (albeit more time-consuming) approach. The specific procedure is as follows. We start with a pre-trained model, which consists of all the full-resolution weights and perform a nearest rounding quantization on the complex weights. After quantization, the weights are divided into two groups, one of them will be retrained and the other group not. This partition of the weights in groups is done not once, but several times in parallel by random selection (the number of parallel weights partitions is chosen from 2 to 20 depending on the number of residual weights to be retrained). For each partition, after retraining, the weights will be quantized again to evaluate the performance based on the BER on the training data set. By comparing the task performance for these different partitions, we can pick the best one.

We iteratively conduct the procedure above. In each iteration, the ratio of weights to be retrained decreases by a factor of two, starting from an initial value of 0.5. We typically perform 4 iterations, so for our 16-node reservoir, the number of trained weights in each iteration is 8, 4, 2, 1. As the iteration proceeds, we also decrease the number of partitions to be evaluated from 20 in the beginning to 2 at the end, as fewer and fewer weight combinations become available. In order to increase the stability of the procedure, weights that have been selected as fixed in previous iterations remain fixed in future iterations as well.

### Quantization of the optical weighting elements

**Figure 2:**
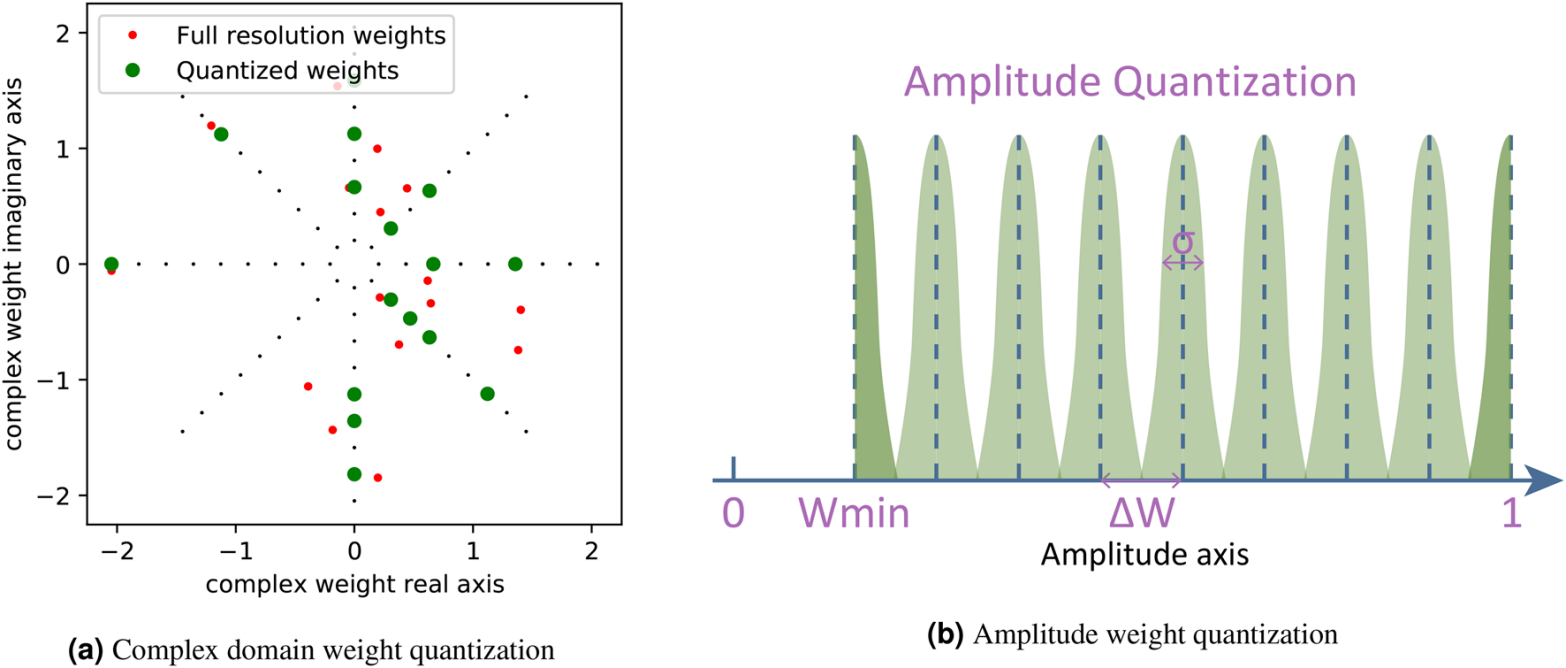
(**a**) An illustration of complex weights quantization in a coherent photonic readout system. Small black dots are all available quantization weights in the complex plane. Red dots represents the pre-trained full resolution weights, Green dots are corresponding quantized weights. Weighting noise in this figure is set to 0. (**b**) An illustration of amplitude quantization model for a general optical weighting element. The green shadowed areas represent noise probability distribution.

Here, we describe in more detail what model has been used for the quantization procedure itself. We aim to provide a high-level model for the weighting elements (both amplitude and phase) that is as generic as possible, without being tied to any specific hardware implementation details. Rather, in the quantization process, we take three major aspects into account: extinction ratio, resolution, and noise. Figure 2a shows the quantization of complex weights in the complex plane, without weighting noise.

#### Amplitude quantization

The readout systems that we model here are supposed to be an optical system without amplification. In this case, the largest weight of the amplitude will be 1 (light passing through without being affected) and the lowest weight will be 0 (light is fully blocked by the weighting elements). However, in a realistic optical weighting element, the fully blocked weight ‘0’ is often not achievable. Most elements have an extinction ratio describing how much optical power it can block compared to the maximum power that can pass through. For example, given an an extinction ratio of 10 and a maximum weight of 1, the minimum weight will be 0.1.

The resolution of the weights is characterized as a number of bits or number of levels. All the possible weighting levels are uniformly distributed within the same interval, taking into account the minimum weight because of the extinction ratio. Figure 2b illustrates this in more detail. The minimum weight the readout system can reach is given by:

$$\begin{aligned} w_{min} = 1/\text {Extinction ratio} \end{aligned}$$

After quantization, weights that are below this level will be rounded up to this value.

The distance between two adjacent weighting values is given by:

$$\begin{aligned} \Delta w = (1 - w_{min}) / (N - 1) \end{aligned}$$

where N is the number of available weighting levels, determined by the resolution of the optical element.

Noise and drift are of course very dependent on the details of the hardware used, but for our purposes, we will abstract the noise as a Gaussian distribution. The noise level numbers we give in the following section are based on the standard deviation $\sigma$ of the Gaussian distribution as follows:

$$\begin{aligned} \text {Noise level} = \sigma / \Delta w \end{aligned}$$

#### Phase quantization

In the phase quantization process, the extinction ratio issue will not play a role, and there will only be resolution and noise. The quantization will be implemented between $[0, 2\pi )$. All the levels are also evenly distributed. Noise is also modeled as Gaussian distribution.

### Regularization

The regularization method used in this work is L2 regularization. In the photonic hardware readout system, there is no significant redundancy of connections or weights, and each weight is realized by a real waveguide through which the optical signals are passing. It is not practical to ’cut’ or ’prune’ any hardware optical connections. Therefore we choose L2 over L1 regularization since we do not want to waste any readout waveguide and L1 tends to result in zero weights. Choosing the best regularization parameter is crucial to prevent overfitting and to help the system to be robust against noise and quantization^27^.

### Baselines

In this paper, we choose two baseline methods to compare our results with. The first is nearest rounding quantization, which is referred to as ‘naive quantization’ in the rest of the paper. The second baseline is randomized rounding quantization^28^, which will be referred to as ‘random rounding’. Random rounding is a reasonable baseline to compare our method too, since it also gives an optimized performance by statistic selection of better weight choices. In our implementation of random rounding, we set the rounding probability based on the distance of a weight to a nearby quantization value. We repeat this procedure 20 times and pick the result with the lowest error rate.

### Training procedure

The original idea of reservoir computing is to use linear (ridge) regression to train the weights of a readout system. This saves a lot of training time compared to using gradient descent, as weighing the reservoir output signals is just a linear regression and can be calculated explicitly. In a coherent photonics reservoir however, the outputs of the reservoir are coherent optical signals containing amplitude and phase information, i.e. complex numbers. In the readout system, these optical signals are mixed together and interfere with each other to produce a final optical signal injected into a photodetector. Therefore the output of such a readout system is a single electrical signal containing only intensity information. This is a nonlinear conversion from a complex-valued optical signal with an amplitude and phase to a purely real intensity. To incorporate such a nonlinear conversion, we can no longer use the one-step solution based on the Moore–Penrose inverse of linear ridge regression, since there is no unique way to choose a phase of the signal before the photodiode that gives rise to the desired amplitude after the photodiode. Therefore, in our learning phase, we optimize the weights with gradient descent, explicitly taking the nonlinear photodiode into account. Using this method gives us more robust results and increases the computation capability of our reservoir system.

Another decision we made is to use the Mean Squared Error (MSE) as a loss function to optimize the optical weights. One could argue to use logistic regression instead, because the tasks we are trying to solve are all classification tasks. However, to build an optical neuromorphic computing system, we have to consider the hardware implementation of each signal processing step. In our hardware system, the output electrical signal from the final photodetector will be used as the final prediction signal without further signal processing. Using logistic regression would assume another layer of nonlinear calculation, the sigmoid function. This process would result in a more complex integration technology and induce more latency and energy cost.

## Results

In this section, we present simulation results for the performance of the proposed method on two tasks: header recognition and a boolean XOR operation. The bit sequences will be encoded to optical signals before the simulation. The architecture we choose for these tasks is the 4-port swirl reservoir network^29^ with 16 nodes. We train all the tasks on fully integrated passive photonic reservoirs with optical integrated readout systems. We treat the full resolution weighting performance with weighting noise as the idealized best performance the system can achieve, and also compare our method with the performance of ‘naive’ quantization and random rounding quantization.

The simulation consists of two parts. The first one is to generate the reservoir signal. We use Caphe^30^ to simulate the reservoir circuit with subsampling of 20 points per bit on the intensity-modulated input signal. The delays in the photonic reservoir are optimized for an input signal speed of 32 Gbps. The response of the photonic reservoir will be the complex output signals from each of the nodes, consisting of amplitude and phase information. The second part is the training and quantization of the weights using our proposed method. We use PyTorch^31^ to implement this.

### Header recognition

The header we use in the header recognition task is a 4-bit header ‘1101’. We want the final readout signal to be ‘1’ whenever the chosen header appears in the input signal, and ‘0’ otherwise. The reservoir architecture is a $4\times 4$, 16 node reservoir. Although header recognition can be solved in a multi-class classifier, the optical readout hardware we use here is not suited for multi-channel optical output. As mentioned, we use an MSE loss function with L2 regularization and train our models with gradient descent using Adam^32^.

We now discuss the influence of resolution, noise and extinction ratio on the performance. For the following figures indicating the performance in terms of BER, error bars represent the standard deviation of the results from 10 different reservoirs. It indicates the performance consistency of our method over different reservoir chips with random fabrication imperfections.

**Figure 3:**
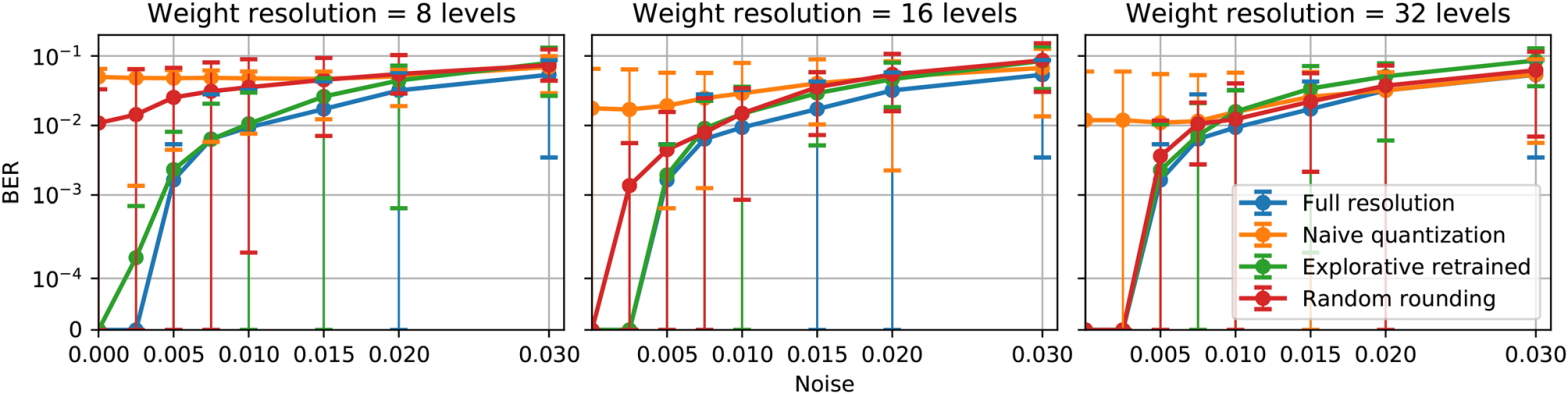
Performance comparison of three different weighting resolutions, 8 levels (left), 16 levels (middle), 32 levels (right) for the 4-bit header recognition task over different noise levels. The blue curve represents the performance of full-resolution weights; the orange curve represents ‘naive’ quantization weights; the green curve for explorative retrained quantization weights.

#### Noise

Noise has a very significant influence on performance. Figure 3 shows the performance evaluation of the Bit Error Rate (BER) at different noise levels at 8-level, 16-level and 32-level resolution on the 4-bit header recognition task. The top figure with 8-level resolution shows that overall, the quantized weights obtained by our explorative retraining method consistently provide performances very close to the full resolution weights. For more substantial noise levels of the optical weighting elements, as shown in the far right part of the graphs, the BER increases significantly, which reflects the severe impact that the noise of the optical weighting elements brings to system performance, even for a full-resolution system. Apart from those situations of large noise levels, the retrain method is performing well throughout and gives several orders of magnitude better BER than the ‘naive’ quantization of the weights where the readout is trained and quantized only once. When there is no noise on the weights, the retrain method gives almost the same performance on the limited resolution readout system compared to a full resolution readout system. This result is rather surprising, given that the resolution here is only 8 levels. The random rounding method also fails to deliver a reasonable performance at these levels of resolution.

For higher resolutions, namely 16 levels and 32 levels, the explorative retraining method provides even closer performance to the full resolution readout systems. As the resolution increases, the ‘naive’ quantization method also achieves lower bit error rates, however still not comparable to the full resolution and explorative retrained weights.Random rounding only provides comparable performance to the full resolution weights at 32-level resolution. For lower resolutions, its performance is considerably worse. When the noise levels are high, all four sets of weighting strategies are giving a similar performance (especially at 16 and 32 levels resolution all four results are indistinguishable), The reason is that, as the resolution increases, the interval between two adjacent weighting level is in the same range of the variance of the noise. These three weighting methods will provide similar weighting values statistically.

**Figure 4:**
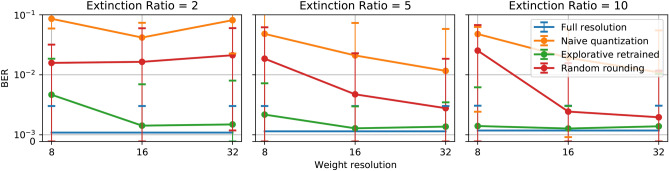
Performance comparison of three different extinction ratio, 2 (left), 5 (middle), 10 (right) for the 4-bit header recognition task over different resolutions. The blue curve represents the performance of full-resolution weights; the orange curve represents ‘naive’ quantization weights; the green curve for explorative retrained quantization weights.

#### Extinction ratio

Figure 4 illustrates the influence of the extinction ratio of the optical weighting elements. The figure shows that for directly quantized weights, a higher resolution always gives better BER regardless of the extinction ratio of the weighting components. However, when the extinction ratio gets higher, the performance from the 32-level ‘naive’ quantized weights and random rounding weights are closer to the full resolution weights. This result is intuitive since higher extinction ratio means the weight range is more preserved after quantization, and when the extinction ratio is low, more initially lower weights will be rounded up to the lowest weighting value that the extinction ratio allows. In this task, extinction ratios of 5.0 and 10.0 do not give much difference compared to the huge performance drop for the extinction ratio of 2.0. For photonics weighting components, an extinction ratio of 5.0 to 10.0 is not a very strict requirement, and therefore photonics reservoir hardware systems should be able to deliver the required extinction ratio for this task.

In contrast, the retraining method gives consistently better performance because the weights are continually evolving to adapt to the weighting range that the extinction ratio defines. The performance levels are always very close to those of the full resolution, with a limited impact from the extinction ratio.

#### Training time

The training time of the explorative method takes longer due to iterations of training and weight connection selecting. For the header recognition task, training the 16-node photonic reservoir system using nearest rounding takes around 2 minutes, with CUDA enabled on a Nvidia GeForce GTX 970 GPU. The explorative training procedure takes around 18 minutes and random rounding method takes 5 minutes. For a reservoir that has more nodes, the training time does not scale up quickly with the number of nodes, since the training utilizes the parallel processing of the GPU. Moreover, as mentioned, the number of nodes in a photonics reservoir is usually below one hundred to control optical loss.

Although it is a more time-consuming training procedure, the extra training time does not impact to the run time the system after programming. As mentioned, the training of the system is done off-line. Once the system is trained, the photonic reservoir computing works in the all-optical domain without any optical-electrical conversion and can have an extremely high bandwidth. Therefore the off-line training time does not result in any runtime latency.

### 4-Bit delayed XOR

We chose 4-bit delayed XOR (i.e. calculating the XOR of the current bit and 4 bits ago) as our second task. The reason is that XOR is a more nonlinear task compared to the header recognition, so it is interesting to see how it behaves under weight quantization situations.

**Figure 5:**
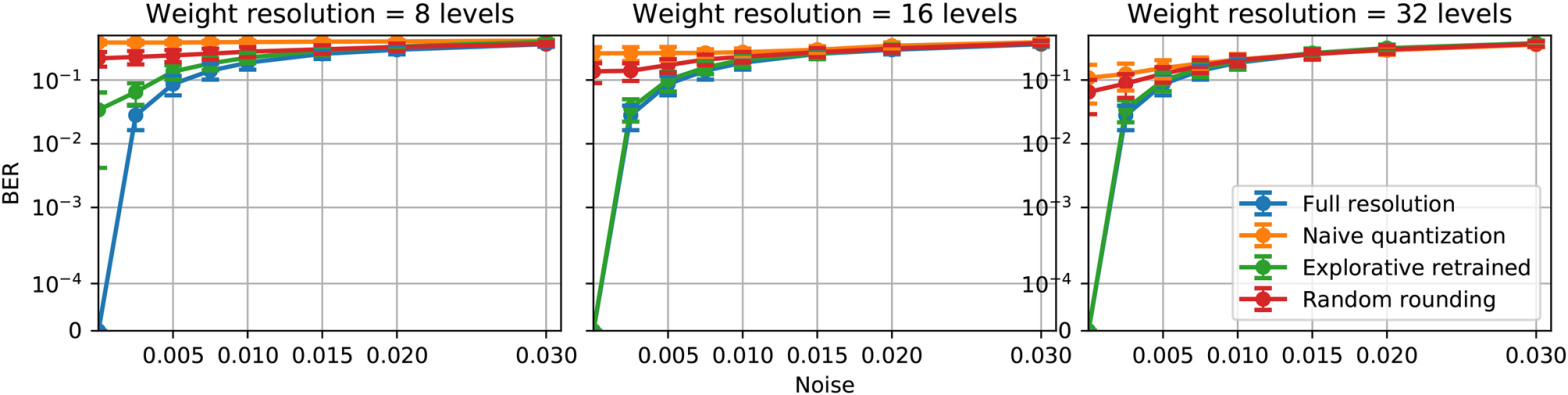
Performance comparison of three different weighting resolutions, 8 levels (left), 16 levels (middle), 32 levels (right) for the 4-bit delayed XOR task vs different noise levels. The blue curve represents the performance of full-resolution weights; the orange curve represents ‘naive’ quantization weights; the green curve for explorative retrained quantization weights.

Figure 5 presents the performance of our quantization retraining method on low-precision weights (8, 16, 32 levels) at different noise levels. From the blue curve, it can be seen that 4-bit delayed XOR is indeed a harder task to tackle since even with full precision, the BER increases significantly with a small amount of noise. Moreover, the task is also very sensitive to low precision weights, as shown from the ‘naive’ quantization weights (orange curves), at the 8 levels resolution, the tasks is unsolvable even without any noise involved. In the meantime, we also observe that our explorative retrained weights are capable of providing very close performance to full resolution weights, except for 8 levels resolution, where due to the extra nonlinearity requirement of the task, retrained weights find themselves hard to follow. But still, ‘naive’ direct quantized weights and random rounding quantized weights are severely outperformed by our explorative retraining method at the overall spectrum of the different noise levels.

**Figure 6:**
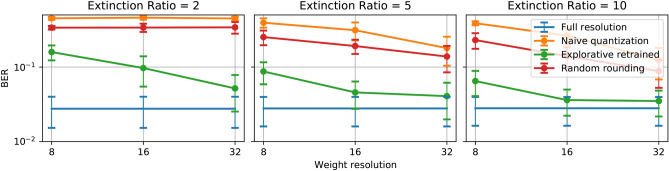
Performance comparison of three different extinction ratios, 2 (left), 5 (middle), 10 (right) for the 4-bit delayed XOR task over different resolutions. The blue curve represents the performance of full-resolution weights; the orange curve represents ‘naive’ quantization weights; the green curve for explorative retrained quantization weights.

The performance dependence on resolution and extinction ratio is shown in Fig. 6. The noise level here is 0.0025. Similar to the header recognition task, a higher extinction ratio provides better direct quantization performance. Weighting elements with an extinction ratio of 2 are not able to provide enough weighting range for direct quantized weights to deliver workable performance regardless of the weighting resolution. The explorative retraining method is also affected at the 8 levels resolution. When the extinction ratio is 10, the quantization retraining method delivers performance close to the full resolution, from which we can draw a statement that an extinction ratio of 10 is sufficient for this task. For random rounding weights, it always delivers slightly better results compared to the ’naive’ quantization method. However, it is outperformed by the explorative retraining method by a big margin, especially in higher resolutions regardless of extinction ratios.

## Conclusion

In this paper, we addressed the limited weight resolution in an integrated all-optical readout system for photonic reservoir computing systems. A high-level general modeling for realistic integrated optical weighting elements enables us to characterize the influence of the weighting resolution, noise, and extinction ratio on the system performance. Our proposed explorative retraining method focused on identifying the best weights to be retrained. It is shown that in situations where both the number of output channels and the weighing resolution is extremely limited, our proposed method still delivers performance very close to that of full resolution weights over a large span of weighting noises.
